# Spatiotemporal Expression Patterns of Critical Genes Involved in FGF Signaling During Morphogenesis and Odontogenesis of Deciduous Molars in Miniature Pigs

**DOI:** 10.7150/ijms.61798

**Published:** 2022-01-01

**Authors:** Wenwen Guo, Xiaoyu Lin, Ran Zhang, Lei Hu, Jiangyi Wang, Fu Wang, Jinsong Wang, Chunmei Zhang, Xiaoshan Wu, Songlin Wang

**Affiliations:** 1 Beijing Laboratory of Oral Health; Capital Medical University School of Stomatology, Beijing, China.; 2 Department of Oral Pathology, Peking University School and Hospital of Stomatology, Beijing, China.; 3 Department of Oral Basic Science, School of Stomatology, Dalian Medical University, Dalian, China.; 4 Department of Biochemistry and Molecular Biology, Capital Medical University School of Basic Medical Sciences, Beijing, China.; 5 Department of Oral and Maxillofacial Surgery, Xiangya Hospital, Central South University, Changsha, China.; 6 Academician Workstation for Oral-Maxillofacial Regenerative Medicine, Central South University, Changsha, China.

**Keywords:** miniature pig, tooth development, deciduous molar, fibroblast growth factor, dental epithelium, dental mesenchyme

## Abstract

The fibroblast growth factor (FGF) pathway plays an important role in epithelial-mesenchymal interactions during tooth development. Nevertheless, how the ligands, receptors, and antagonists of the FGF pathway are involved in epithelial-mesenchymal interactions remains largely unknown. Miniature pigs exhibit tooth anatomy and replacement patterns like those in humans and hence can serve as large animal models. The present study investigated the spatiotemporal expression patterns of critical genes encoding FGF ligands (*FGF3*, *FGF4*, *FGF7*, and *FGF9*), antagonists (*SPRY2* and *SPRY4*) and receptors (*FGFR1*, *FGFR2*, and *FGFR3*) in the third deciduous molars of miniature pigs at the cap (embryonic day 40, E40), early bell (E50), and late bell (E60) stages. The results of *in situ* hybridization (ISH) with tyramide signal amplification and of qRT-PCR analysis revealed increased expression of *FGF7*, *FGFR1*,* FGFR2*, and *SPRY4* in dental epithelium and of* FGF7* and *FGFR1* in mesenchyme from E40 to E50. In contrast, the results revealed decreased expression of *FGF3*,* FGF4*,* FGF9*, and* FGFR3* in dental epithelium and of *FGF4*, *FGF9*, *FGFR2*, and *FGFR3* in the mesenchyme from E40 to E60. Mesenchyme signals of *FGF3*, *FGF4*, *FGF7*, *SPRY2*, *FGFR2*, and *FGFR3* were concentrated in the odontoblast layer from E50 to E60. The distinct expression patterns of these molecules indicated elaborate regulation during dental morphogenesis. Our results provide a foundation for further investigation into fine-tuning dental morphogenesis and odontogenesis by controlling interactions between dental epithelium and mesenchyme, thus promoting tooth regeneration in large mammals.

## Introduction

Tooth development begins when the local oral epithelium thickens to form a placode called the dental lamina, which invaginates to surround the underlying mesenchyme and form a follicle that enters the bud stage. At the cap stage, the tip of the bud invaginates and the dental epithelium differentiates into the enamel organ, which includes inner (IEE) and outer (OEE) enamel epithelium, stellate reticulum (SR), and an intermediate cell layer. The apical part of the IEE and the underlying dental mesenchyme (DM) differentiate into ameloblasts and odontoblasts at the late bell stage 1. Reciprocal interactions between the ectoderm-derived epithelium and underlying neural crest-derived mesenchyme occur at every step of development 1, 2.

Proteins in the fibroblast growth factor (FGF) family mediate inductive interactions between dental epithelium (DE) and the DM during successive stages of tooth formation 3. Based on sequence similarity, receptor specificity, and binding affinity, FGFs can be subdivided into the following subfamilies: FGF1, FGF4, FGF7, FGF8, FGF9, FGF11, and FGF15 4-7. Most FGFs mediate their biological responses as extracellular proteins that bind to and activate cell surface tyrosine kinase FGF receptors (fibroblast growth receptors; FGFRs) 1-4 4.

Fibroblast growth factor signaling is crucial during the development of tooth epithelium and mesenchyme 8. The initial expression of *Fgf4*,* Fgf8*, and *Fgf9* in the dental epithelium and later in the enamel knot affects cell proliferation during tooth initiation and subsequent morphogenesis that regulates the establishment of tooth shape 9-14. Furthermore, *Fgf3*, *Fgf7*, and *Fgf10* are expressed in the DM of mice 15.

Sprouty proteins act downstream of FGF signal activation by interfering with the RAS-MAPK pathway. Sprouty RTK signaling antagonist (*SPRY*) genes were originally identified as antagonists of FGFR-mediated signaling 16, 17. The expression of the *Spry1*, *Spry2*, and *Spry4* genes encoding antagonists is required for appropriate molar cusp patterning throughout enamel development 13, 18, 19. However, whether joint FGF and SPRY expression regulate tooth development by modulating epithelial-mesenchymal interactions in large mammals remains unclear.

Tooth development and the cascade initiation of permanent molars have been investigated in the third deciduous molars (DM3) of miniature pigs 20-22. Here, we characterized the dynamic expression profiles of genes associated with the FGF signaling pathway in DM3 of miniature pigs at the cap, early, and late bell (secretory) stages, with focus on associations with cusp patterning and tooth mineralization. Our findings clarified interactions between the epithelium and mesenchyme during tooth development, which might be applicable to human tooth regeneration.

## Results

### Morphology of DM3 across developmental stages in miniature pigs

**Figure 1:**
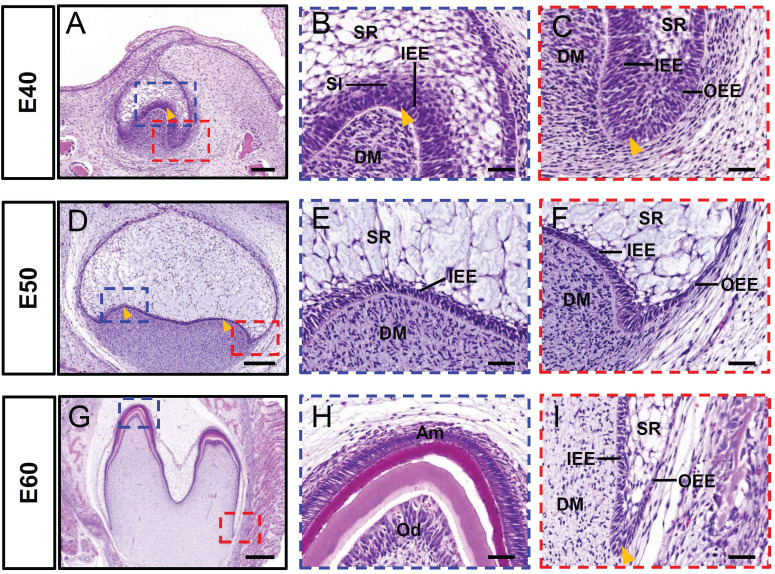
** Morphology of DM3 across developmental stages in miniature pigs.** (A-I) HE staining at different stages during DM3 tooth germ morphogenesis. Boxed regions in A, D, G are magnified in B-C, E-F, H-I. (A-C) Tooth germ progresses to cap stage at embryonic day 40 (E40). Arrowheads, primary enamel knot in A and B, and cervical loop in C. (D-F) At E50, DM3 reaches early bell stage and secondary enamel knots (arrowheads) begin to form; (G-I) At E60, DM3 germ developed into late bell (secretory stage), with elongated cervical loop (arrowhead). Parts of IEE and dental papilla cells at cusp tip have differentiated into ameloblasts and odontoblasts. Am, ameloblasts; DM, dental mesenchyme; DM3, third deciduous molars; HE, hematoxylin and eosin; IEE, inner enamel epithelium; Od, odontoblasts. OEE, outer enamel epithelium; SR, stellate reticulum; SI, stratum intermedium. Scale bars: 100, 200, and 500 μm in A, D, and G, respectively, and 50 μm in B-C, E-F, H-I.

The cap, early bell, and late bell (secretory) stages of DM3 development occurred in miniature pigs at embryonic day 40 (E40), E50, and E60, respectively. The tooth bud of DM3 entered the cap stage at E40 (Figure 1A). This was characterized by formation of the primary enamel knot, a central region of epithelium (arrowhead) that comprises the signaling center involved in regulating tooth shape (Figure 1B). Furthermore, the epithelial bud underwent specific folding (arrowhead) and was composed of IEE and OEE, SR, and stratum intermedium (Figure 1B-C). At this stage, the cervical loop was formed by the IEE and OEE, together with the SR (Figure 1C). A typical feature of E50 was the formation of secondary enamel knots (arrowheads) and the start of transformation into a bell shape (Figure 1D-F). The DM3 transitioned to the later bell stage at E60 (Figure 1G). The cells at the tip of the IEE differentiated into ameloblasts and began to form pre-enamel prisms, whereas the cells at the tip of the DM differentiated into odontoblasts (Figure 1H). Furthermore, the cervical loop (arrowheads), formed by the IEE and OEE, with a minor contribution from the SR, elongated toward the direction of the future root (Figure 1I). Thus, the analysis of DM3 development at stages E40-E60 revealed information about the processes of cusp shaping, crown mineralization, and root morphogenesis in miniature pigs.

### Dynamic expression of genes encoding FGF ligands and antagonists during morphogenesis of DM3

**Figure 2:**
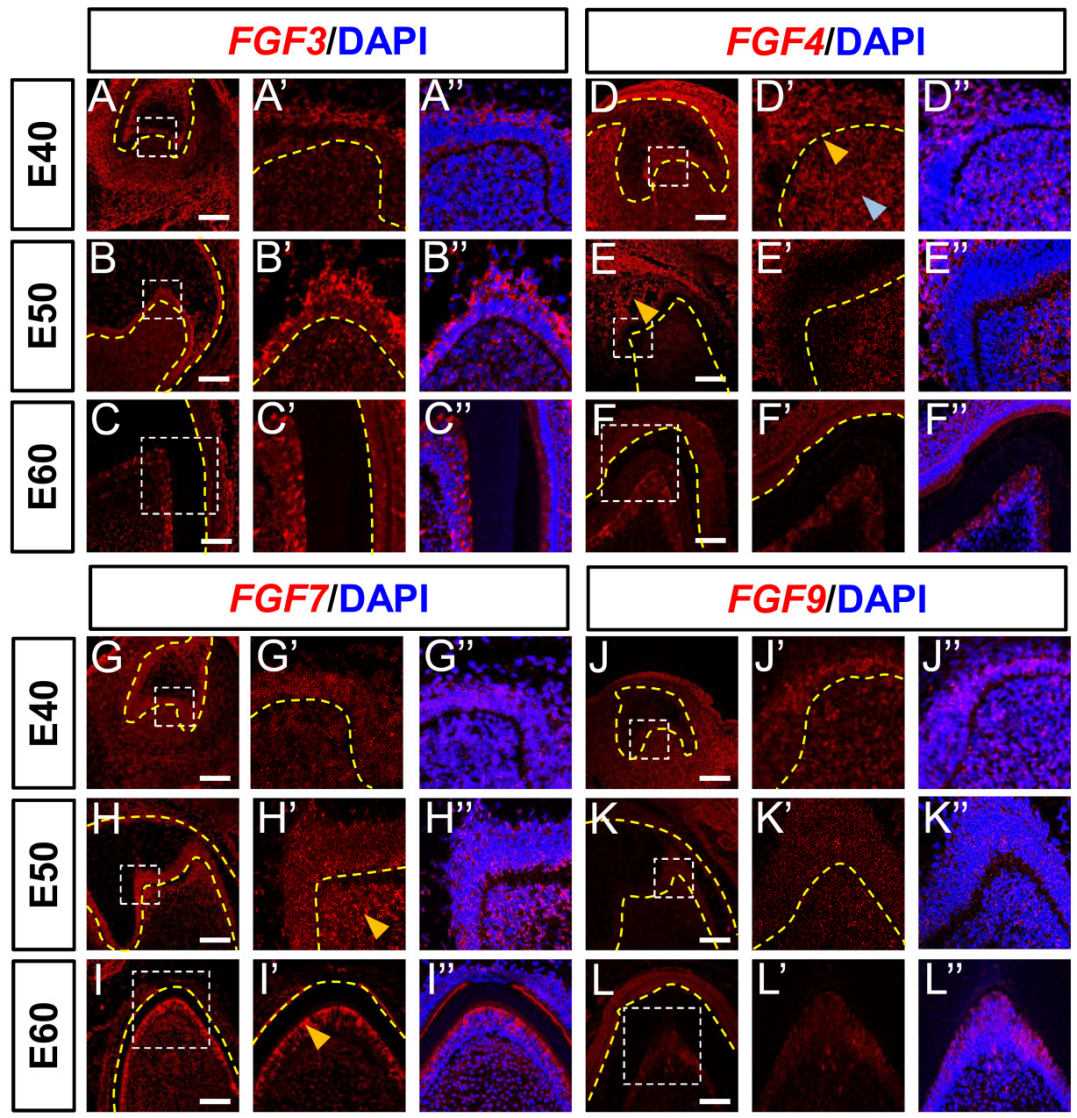
**Dynamic expression of genes encoding FGF ligands during morphogenesis of DM3.** (A-L)* In situ* hybridization (ISH) shows mRNA expression of FGF ligands (red) and nuclei stained with DAPI (blue) from E40 to E60. White boxed regions in A-L are magnified in A'-L', and DAPI-stained nuclei are overlaid in A”-L”. Expression of *FGF3* (A-C) and *FGF4* (D-F) mRNA from E40 to E60. Yellow and blue arrowheads, enamel knot and mesenchyme, respectively in D'. Yellow arrowhead, SR in E. (G-I) Expression of *FGF7* mRNA from E40 to E60; Yellow arrowheads, apical mesenchyme in H', and odontoblasts in I'. (J-L) Expression of *FGF9* mRNA E40 to E60. Yellow dotted line, boundary of tooth epithelium and mesenchyme. Scale bar, 100 μm. E, epithelium; SR, stellate reticulum.

The *Fgf3*,* Fgf4*, *Fgf7* and *Fgf9* mRNAs are central components of the typical FGF signaling pathway, whereas *Spry2* and *Spry4* are key antagonists 13. Therefore, we examined the expression of these genes in the epithelium and mesenchyme of DM3 at E40, E50, and E60 using *in situ* hybridization (ISH) and immunofluorescence staining. We identified *FGF3* mRNA mainly in the IEE, SR, and OEE at E40 (Figure 2A), and the apical site of the IEE became more obvious at E50, indicating that FGF3 plays a role in cusp patterning (Figure 2B). The expression of *FGF3* in the DM was low at E40, but gradually increased from E50 to E60 and finally localized in the odontoblast cell layer at E60 (Figure 2B and C), whereas* FGF3* expression in the cervical loop increased from E40 to E50 and E60 (Figure S2A-C). We found that *FGF4* mRNA was expressed in the SR, enamel knot (yellow arrow), and DM (blue arrow) at E40 (Figure 2D). However, expression at E50 was increased in the SR (yellow arrow), but decreased in the mesenchyme and finally localized at the odontoblast layer at E60 (Figure 2E and F). The expression in the cervical loop increased at E50 but decreased at E60 (Figure S2D-F).

We detected *FGF7* mRNA in both DE and DM at the cap stage (E40; Figure 2G). Mesenchymal expression of *FGF7* mRNA was elevated apically at E50 (yellow arrow) and concentrated in the odontoblast layer (yellow arrow) at E60 (Figure 2H and I). The apical restriction and concentration indicated that FGF7 regulates odontoblast differentiation. In addition, *FGF7* expression in the cervical loop was reduced from E40 to E60 (Figure S2G-I). Although *FGF9* mRNA was mainly expressed in the IEE and DM at E40 (Figure 2J), the abundance was obviously reduced at E50 and E60 (Figure 2J-L). We found *FGF9* mRNA in the cervical loops at E40 and E50 (Figure S2J-L). In summary, *FGF3* and *FGF7* mRNA expression was upregulated and concentrated in the mesenchyme from E40 to E60. In contrast, *FGF4* and *FGF9* mRNA expression in the mesenchyme decreased from E40 to E60.

**Figure 3:**
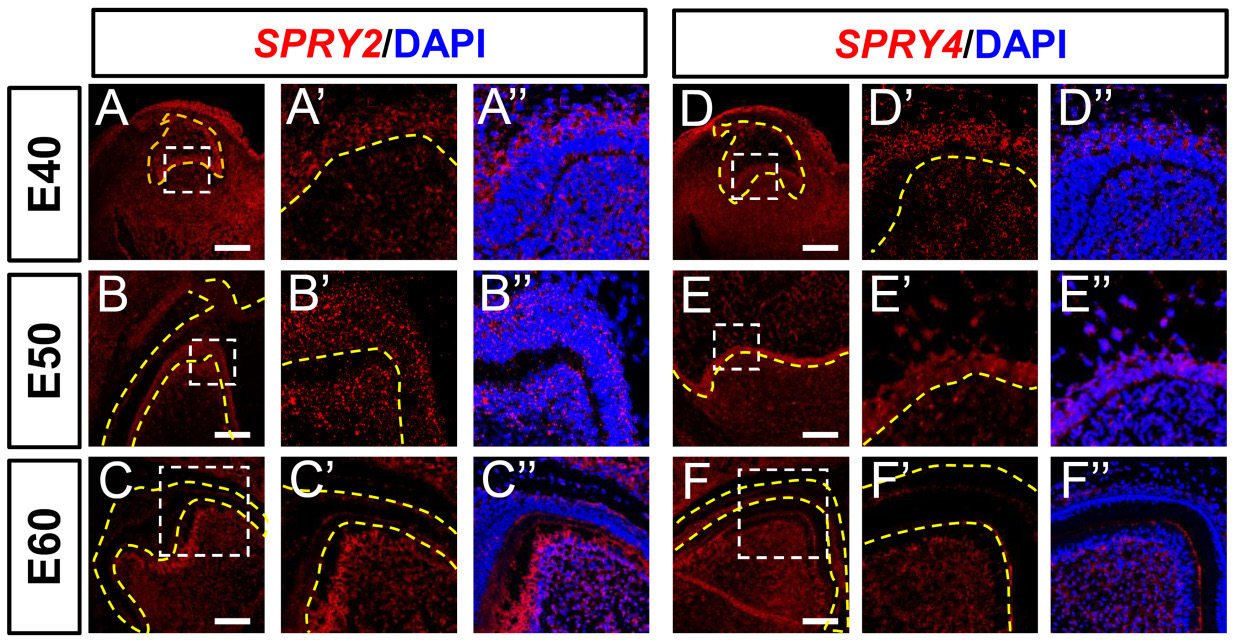
** Dynamic expression of genes encoding FGF antagonists during morphogenesis of DM3.** (A-F)* In situ* hybridization (ISH) shows mRNA expression of FGF antagonists (red) and nuclei stained with DAPI (blue) from E40 to E60. White boxed regions in A-F are magnified in A'-F', and DAPI staining is overlaid in A''-F”. Expression of *SPRY2* (A-C) and *SPRY4* (D-F) mRNA from E40 to E60. Yellow dotted line, boundary of tooth epithelium and mesenchyme. Scale bar, 100 μm.

We explored the expression of FGF signaling antagonists. The expression of *SPRY2* was abundant in the DE at E40, increased at E50, and decreased at E60 (Figure 3A-C). The expression of *SPRY2* was increased in the DM, and concentrated in odontoblasts from E40 to E60 (Figure 3A-C). The expression of *SPRY4* was detected in the intermediate cell layer at E40, and increased at E50, but decreased in the IEE at E60 (Figure 3D-F). We detected *SPRY4* mRNA in DM from E40 to E60 (Figure 3E-F). Moreover, *SPRY2* and* SPRY4* mRNAs were expressed in the cervical loop during the three stages (Figure S3A-C and S3D-F). In summary, *SPRY2* and *SPRY4* were upregulated in DE, whereas only *SPRY2* was upregulated in the DM during odontogenesis.

### Distribution of FGF ligand proteins during morphogenesis of DM3

**Figure 4:**
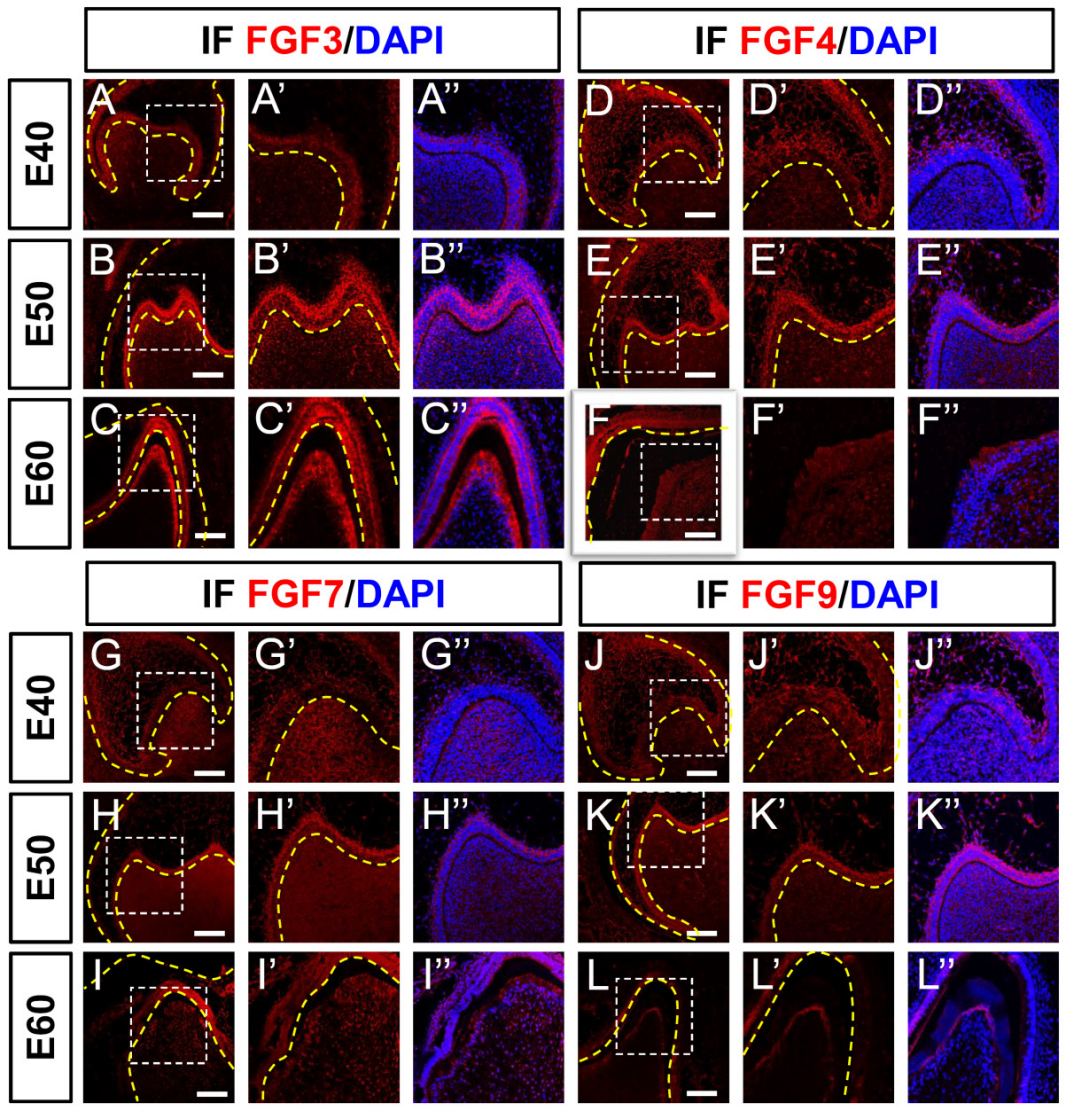
** Protein expression of FGF ligands during morphogenesis of DM3.** (A-F) Immunofluorescence (IF) staining shows protein expression of FGF ligands (red) and nuclei stained with DAPI (blue) from E40 to E60. White boxed regions in A-L are magnified in A'-L', and overlaid with DAPI staining in A'-L”. Expression of (A-C) FGF3, (D-F) FGF4, (G-I) FGF7 and (J-L) FGF9 from E40 to E60. Yellow dotted line, boundary of tooth epithelium and mesenchyme. Scale bar, 50 μm.

We examined the distribution of several FGF proteins at the three stages of DM3 development using immunofluorescence staining to confirm the results of ISH. We found that FGF3 protein was expressed mainly in the DE at E40, in both the DE and DM at E50, and in the ameloblast and odontoblast layer at E60 (Figure 4A-C). The expression of FGF3 protein was abundant in the cervical loop at E50 and E60 (Figure S4A-C). The expression patterns of FGF3 protein and mRNA were similar.

Although FGF4 protein was expressed mainly in the SR (Figure 4D) at E40, *FGF4* mRNA was simultaneously expressed in the enamel knot and mesenchyme (Figure 2D). By E50, FGF4 was expressed mainly in DE (Figure 4E), whereas *FGF4* mRNA was found primarily in the SR and mesenchyme, but not in the DE at E50 (Figure 2E). At E60, FGF4 protein expression was considerably reduced (Figure 4F), which was similar to the mRNA profile (Figure 2F). The FGF4 protein and *FGF4* mRNA were similarly expressed in the cervical loop from E40 to E60 (Figures S4D-F and S2D-F).

The expression of FGF7 protein and *FGF7* mRNA gradually increased from E40 to E60 in the DE and DM (Figures 4G-I and 2G-I), respectively. The expression of FGF7 protein and *FGF7* mRNA in the cervical loop was weak from E40 to E60 (Figures S4G-I and S2G-I).

The expression level of FGF9 protein (Figure 4J-L) gradually decreased like that of *FGF9* mRNA (Figure 2J-L) in the DE and DM from E40 to E60. The FGF9 protein (Figure S4J-L) and *FGF9* mRNA (Figure S2J-L) were both expressed in the cervical loop from E40 to E60. The different expression levels of mRNA and protein between FGF4 and FGF7 indicated protein translocation between the epithelium and mesenchyme during tooth development.

### Dynamic expression of genes encoding FGF receptors during morphogenesis of DM3

**Figure 5:**
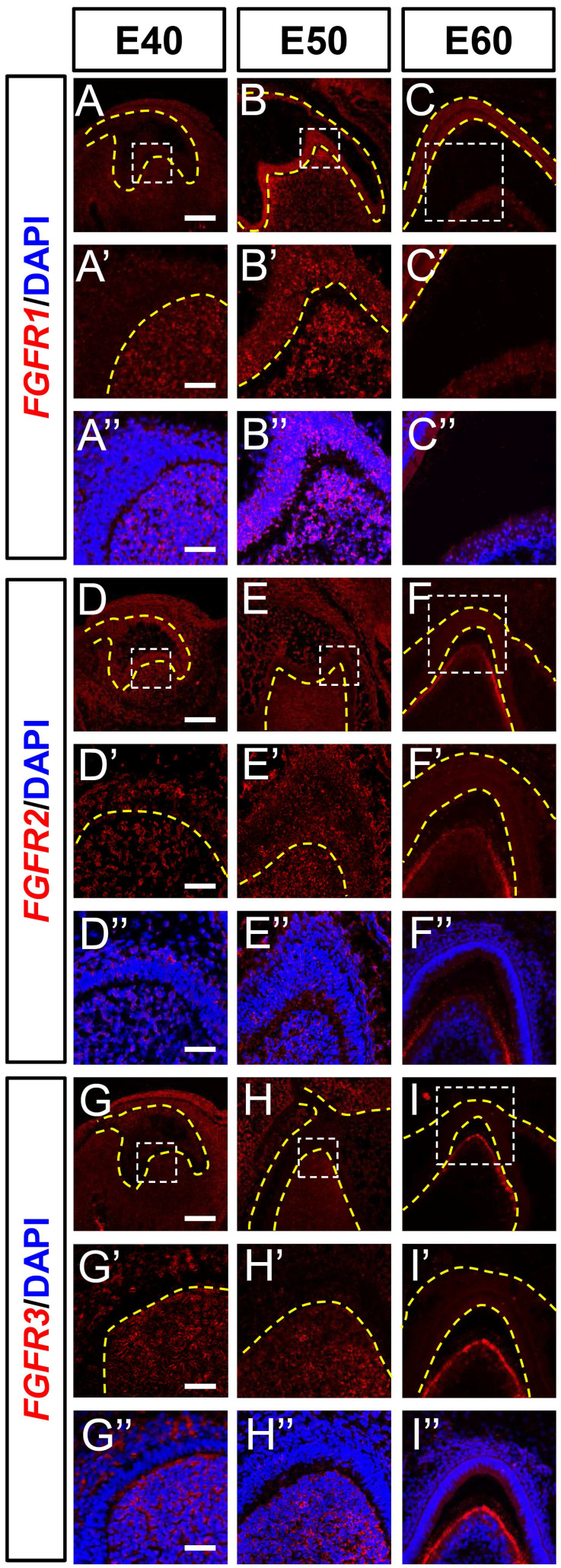
** Dynamic expression of genes encoding FGF receptors during morphogenesis of DM3.** (A-I)* In situ* hybridization (ISH) shows mRNA expression of FGF receptors (red) and nuclei stained with DAPI (blue) from E40 to E60. White boxed regions in A-I are magnified in A'-I', and DAPI staining is overlaid in A'-I”. Expression of (A-C) *FGFR1*, (D-F) *FGFR2*, and (G-I) *FGFR3* mRNA from E40 to E60. Yellow dotted line, boundary of tooth epithelium and mesenchyme. Scale bar, 100 μm.

Fibroblast growth factor ligands bind to FGF receptors on the surfaces of target cells to transfer signals to nuclei. We examined *FGFR1*, *FGFR2*, and *FGFR3* mRNA expression levels in DM3 at the three developmental stages using ISH. The *FGFR1* mRNA was expressed mainly in the DM at E40 and in both DE and DM at E50. Expression was elevated in the epithelium and mesenchyme from E40 to E50 (Figure 5A and B) but greatly reduced at E60 (Figure 5C). The expression of *FGFR1* was abundant at E50 but scarce at E40 and E60 (Figure S5A-C).

Although* FGFR2* was found in the DE and DM at E40 and E50, it was mainly localized in the odontoblast layer at E60 (Figure 5D-F), and *FGFR3* was detected in the SR and DM at E40 (Figure 5G). The expression was restricted to the DM between E50 and E60 and limited to secretory odontoblasts at E60 (Figure 5H and I). Although *FGFR2* and *FGFR3* transcripts were identified in the cervical loop at E40, they were basically absent at E50 and E60 (Figure S5D-I).

### Quantitative gene expression dynamics related to the FGF pathway and its antagonists in DM3

To further understand the expression dynamics of FGF-related genes and antagonists, we quantified their mRNA levels by real-time PCR and analyzed differences among the genes in DE and DM across the three stages. Tooth germs of DM3 were harvested, and epithelium including IEE, OEE, and SR was enzymatically separated from the mesenchyme using dispase II before reverse transcription 26.

**Figure 6:**
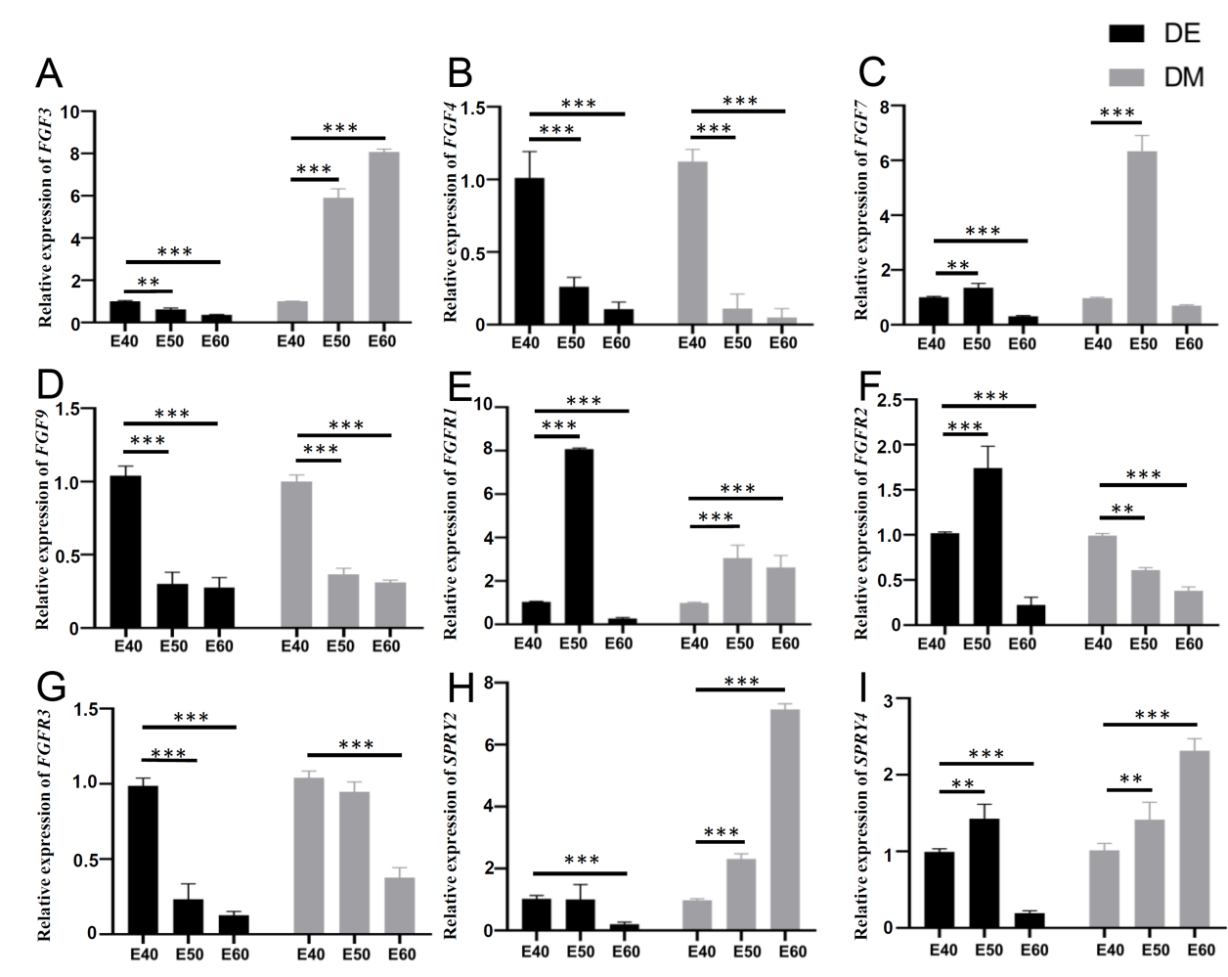
** Quantitative gene expression dynamics related to the FGF pathway and its antagonists of DM3.** (A-I) Relative mRNA expression in dental epithelium (DE) and mesenchyme (DM) from E40 to E60 determined by qRT-PCR. Expression of (A-D) *FGF3*, *FGF4*,* FGF7*, and *FGF9,* and (E-G) *FGFR1*,* FGFR2*, and* FGFR3* from E40 to E60. (H-I) Expression of *SPRY2* and* SPRY4* from E40 to E60. *p < 0.05, **p < 0.01, ***p < 0.001.

The RT-PCR results showed the expression of *FGF3*,* FGF4*,* FGF9*, and* FGFR3* was decreased in the DE from E40 to E60. The expression of* FGF7*, *FGFR1*,* FGFR2*, and *SPRY4* was increased in the DE from E40 to E50, but decreased in the DE from E50 to E60. The results showed the increased expression of *FGF3*,* SPRY2*, and* SPRY4* and decreased expression of *FGF4*, *FGF9*, *FGFR2*, and *FGFR3* in the DM from E40 to E60. The expression of* FGF7* and *FGFR1* was increased in the DM from E40 to E50, but decreased in the DM from E50 to E60 (Figure 6). The trends fit with the ISH results generally.

## Discussion

**Figure 7:**
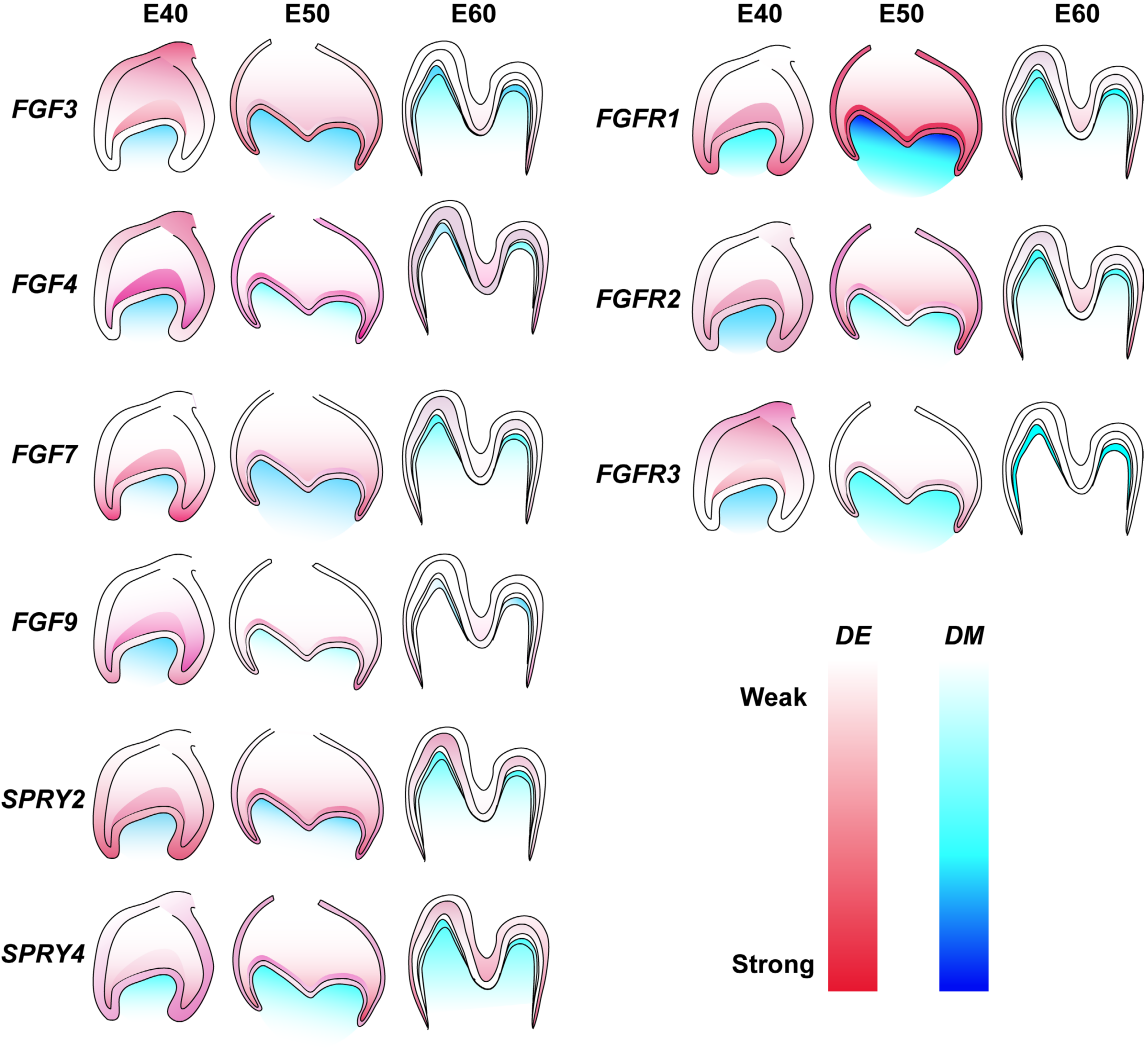
** Gene expression dynamics of FGF pathway and antagonists of DM3.** Lighter and darker shades indicate decreased and increased expression, respectively. DE, dental epithelium; DM, dental mesenchyme.

The role of FGF signaling in the tooth development of miniature pigs is not understood in detail. The present study examined the dynamic expression of representative FGF ligands and receptors in addition to DM3 antagonists at E40, E50, and E60. We found increased expression of *FGF7*, *FGFR1*,* FGFR2*, and *SPRY4* in dental epithelium and of* FGF7* and *FGFR1* in mesenchyme from E40 to E50. In contrast, the results revealed decreased expression of *FGF3*,* FGF4*,* FGF9*, and* FGFR3* in dental epithelium and of *FGF4*, *FGF9*, *FGFR2*, and *FGFR3* in the mesenchyme from E40 to E60. The mesenchymal signals of *FGF3*, *FGF4*, *FGF7*, *SPRY2*, *FGFR2*, and *FGFR3* were concentrated in the odontoblast layer from E50 to E60. Figure 7 shows details of related gene expression. Overall, the FGF signaling pathway and *SPRY* genes might communicate between the epithelium and mesenchyme to maintain tooth morphology and mineralization.

A comparison of the present, with previous findings revealed similarities and differences among mice 13, 27, pigs, and humans 28 (Table 1). Fibroblast growth factor 3 plays a significant role in the proliferation and morphogenesis of tooth development 13. In humans, *FGF3* is predominantly expressed in mesenchyme, particularly in pre-odontoblast and odontoblast cell layers at the late stage 28. Our findings were similar to these. Although *FGF7* is expressed in DE and DM at the cap stage of human teeth 28, it is not involved in tooth development in mice 12. We found remarkably abundant *FGF7* expression in the DM at the early bell stage, but it was restricted to the odontoblast layer at the secretory stage in the DM3. Both *FGF4* and *FGF9* mRNAs are indispensable for DE in the growth of mouse teeth 3. We detected *FGF4* and *FGF9* expression in the DE and the DM from the cap until the early bell stage of DM3. However, its functional role in DM remains unclear. Our results indicated that *FGF3*,* FGF4*,* FGF7*, and *FGF9* ligands play critical roles in cusp patterning, whereas *FGF3* and *Fgf7* might play fundamental roles in odontoblast differentiation.

Fibroblast growth factor ligands bind to their receptors and activate intracellular signal transduction pathways by inducing receptor phosphorylation during tooth growth. In humans, FGF7 binds specifically to FGFR2, whereas FGF3 and FGF10 bind to both FGFR1 and FGFR2 29, 30. The frequency of supernumerary tooth formation is reduced to 60% and 0% by *Fgfr1* and *Fgfr2* null allele heterozygosity, respectively, in transgenic mice 31, 32. We found increased *FGFR1* expression in IEE and DM from E40 to E50, *FGFR2* expression in DE and DM, and *FGFR3* expression mainly in DM at the three stages of DM3. However, these receptors are associated with gene expression and function networks in miniature pigs and require further analysis.

Various sprouty genes act as antagonists of FGF signaling to ensure the correct morphology and size of developing teeth. Mouse *Spry2* is expressed throughout the epithelium, whereas *Spry4* mRNA accumulates exclusively in the DM to prevent the development of supernumerary teeth 13. In continuously growing mouse incisors, *Spry2* and *Spry4* restrict the differentiation of enamel-secreting ameloblasts to the labial side, thereby allowing asymmetric enamel deposition 33. Here, we found that *SPRY2* was initially expressed in DE, then in DE and DM, and mostly in DM from the cap to the late bell stage in miniature pigs, which differed from *SPRY2* expression in mice 13. As antagonists of FGF signaling, *SPRYs* might form an FGF signaling gradient to precisely regulate tooth morphogenesis and odontogenesis.

Interactions among several FGF family members in the IEE and DM regulate the differentiation of dentin and subsequent dentin formation 34. Particularly, FGF4 and FGF9 expressed in epithelial cells are thought to maintain FGF3 expression in the DM to further regulate epithelial cell proliferation and morphogenesis 35. Specifically, FGF3 and FGF7 stimulate the proliferation of inner and outer enamel epithelial cells, respectively 35, 36. In contrast, the loss of SPRY2 function in the epithelium leads to the formation of diastema tooth buds because *Fgf3* expression in the mesenchyme is sufficient to control the expression of *Shh* and perhaps also *Fgf4* 13. The normal function of *Spry4* in the mesenchyme is to prevent epithelial FGF ligands, including *Fgf4* and *Fgf9,* produced in adjacent M1 tooth germ, by inducing or maintaining *Fgf3* expression in the mesenchyme 13. Based on the localization and relevant expression patterns of *FGFs* and* SPRYs* in the DE and DM of DM3, we predict that a feedback mechanism precisely regulates FGF signaling to mediate interactions between the epithelium and mesenchyme during tooth development, which should be further evaluated.

## Conclusions

We determined the spatiotemporal distribution and potential gradient of FGF and sprouty molecules during odontogenesis in large mammals. Epithelial-mesenchymal interactions might be modulated by fine-tuning the FGF pathway to promote cusp patterning and dental mineralization.

## Supplementary Material

Supplementary figures and tables.

## Figures and Tables

**Table 1:** Comparison of expression of the FGF signaling pathway and antagonists between mouse, pig, and human.

Stage	Tissues/cells	Species		
		Mouse 12, 26	Pig	Human 27
Cap stage	Enamel knot/dental epithelium	*Fgf3, Fgf4, Fgf9, Fgf15, Fgf20, Fgf16, Fg17, Fgfr1Ⅲb, Fgfr1Ⅲc, Fgfr2Ⅲb, Spry2, Spry4*	*FGF3, FGF4, FGF7, FGF9, FGFR1, FGFR2, FGFR3, SPRY2, SPRY4*	*FGF3, FGF4, FGF7, FGF8, FGF9, FGF10, FGFR1, FGFR2, FGFR3*
	Dental papilla	*Fgf3, Fgf10, Fgf16, Fgf17, Fgf18, Fgfr1Ⅲc, Fgfr2Ⅲc, Spry4*	*FGF3, FGF4, FGF7, FGF9, FGFR1, FGFR2, FGFR3, SPRY2, SPRY4*	*FGF3, FGF4, FGF7, FGF8, FGF9, FGF10, FGFR1, FGFR2, FGFR3*
Bell stage	Enamel knot/ dental epithelium	*Fgf4, Fgf9, Fgf16, Fgf20, Fgfr1Ⅲb, Fgfr1Ⅲc,*	*FGF3, FGF4, FGF7, FGF9, FGFR1, FGFR2, SPRY2*	*FGF3, FGF4, FGF7, FGF8, FGF9, FGF10, FGFR1, FGFR2, FGFR3*
	Dental papilla	*Fgf3, Fgf10, Fgf15, Fgfr1IIIb, Fgfr1IIIc, Fgfr2IIIc*	*FGF3, FGF4, FGF7, FGFR1, FGFR2, FGFR3 SPRY2, SPRY4*	*FGF7, FGF8, FGF9, FGFR1 FGFR2, FGFR3*
Secretory stage	Ameloblast/ dental epithelium	*Fgf2, Fgf4, Fgf9, Fgf9, Fgf16, Fgfr1, Fgfr2IIIb*	*FGF3, FGF7, FGFR1, SPRY2*	
	Odontoblast/pre-odontoblast	*Fgfr1IIIb, Fgfr1IIIc*	*FGF3, FGF7, SPRY2, FGFR1, FGFR2, FGFR3, SPRY2, SPRY4*	
	Dental papilla	*Fgf15*	*FGF3, FGFR1, FGFR2, SPRY2, SPRY4*	

## Abbreviations

FGF: fibroblast growth factor
FGFRs: FGF receptors
SPRY: sprouty RTK signaling antagonist
DM3: the third deciduous molar
E40: embryonic day 40
E50: embryonic day 50
E60: embryonic day 60
DE: dental epithelium
DM: dental mesenchyme
IEE: inner enamel epithelium
OEE: outer enamel epithelium
SR: stellate reticulum
Am: ameloblasts
Od: odontoblasts
ISH: *in situ* hybridization
TSA: tyramide signal amplification
IF: immunofluorescence
GAPDH: glyceraldehyde-3-phosphate dehydrogenase
real-time RT-PCR: real-time reverse transcription polymerase chain reaction
