# Supplementary material for: Spatiotemporal Expression Patterns of Critical Genes Involved in FGF Signaling During Morphogenesis and Odontogenesis of Deciduous Molars in Miniature Pigs
Source: Int J Med Sci. 2022 Jan 1;19(1):132–41. doi: 10.7150/ijms.61798 (PMC8692127; doi:10.7150/ijms.61798)
Supplement: Supplementary file 1 — Supplementary figures and tables. [file ijmsv19p0132s1.pdf]

Supplementary files

Table S1 Primers of genes for *in situ* hybridization

Gene name	Accession no.	Sense primer	Antisense primer	Size of PCR product (bp)
FGF3	XM_003122421.2	tattctccggacgctacctg	atctcgtggtccttgtggtc	298
FGF4	XM_003122418.2	cacctaaccgcacactgga	aaagtgggtgaccttcatgg	621
FGF7	XM_021073771.1	ggatcctgccaagtttgctc	cataggaagaaaatgggctgttt	551
FGF9	NM_213801.1	ggacagcccggttttgttaa	gtactttgtccgggtccact	512
FGFR1	XM_005671767.3	agcgacaccacctacttctc	acccgccaagcatgtatact	708
FGFR2	NM_001099924.2	ggtgggaatcgacaaagaga	agaggctgactgaggtccaa	828
FGFR3	XM_021100906.1	ccaccgacaaggagctagag	caccgacaggtccaggtact	1349
SPRY2	XM_001927097.5	atcagagccatccgaaacac	cacagttgtcctcgtcatcg	577
SPRY4	XM_003124009.4	cccttctggacagccgtat	cagaaaggcttgtcgggtct	841

Table S2 Primers of genes for real-time RT-PCR

Gene name	Forward primer	Reverse primer
GAPDH	tcctgggctacactgaggac	ccctgttgctgtagccaaat
FGF3	tattctccggacgctacctg	caccgacacgtaccacagtc
FGF4	gctctatggctcggctttct	tcttgcatcaagctgtccgt
FGF7	gcacaaggcagacaacagac	tcactgacctcttcctatttgttct
FGF9	acggatccgaaaaactaaccca	tgccgtttagtcctcgttcc
FGFR1	gactcctaaccccaccttgc	aggtgtagttgcccttgtcg
FGFR2	aacgattacgggtccatcaa	ccgttcttttccacgtgttt
FGFR3	gacgtgcacaacctcgacta	ccgaaggaccagacatcact
SPRY2	gctcagcacaaacacgagag	aagtgcaaggagtgctcgtc
SPRY4	aagtgcaaggagtgctcgtc	ctcgttggtgcagtggtaga

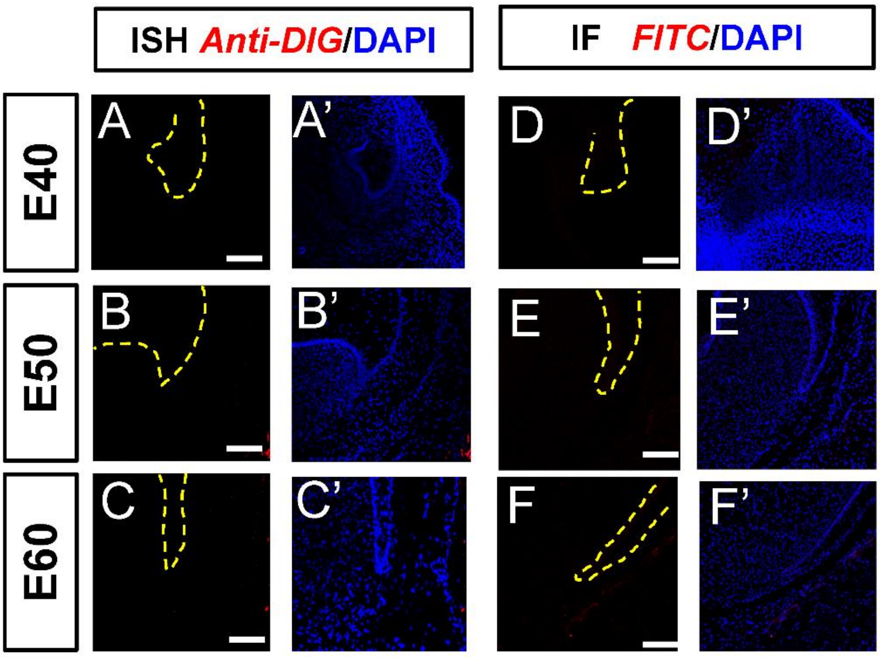

Figure S1. Negative control for the *in situ* hybridization with TSA and immunofluorescence

(A–C) Negative control for the *in situ* hybridization with TSA of E40, E50, and E60; Signals were in red; (A'–C') nuclei stained with DAPI (blue) for A-C; (D–F) Negative control for the immunofluorescence of E40, E50, and E60. Signals were in red; (D'–F') nuclei stained with DAPI (blue) for D-F. The yellow dotted line marked the boundary of tooth epithelium and mesenchyme. Scale bar represents 100 µm.

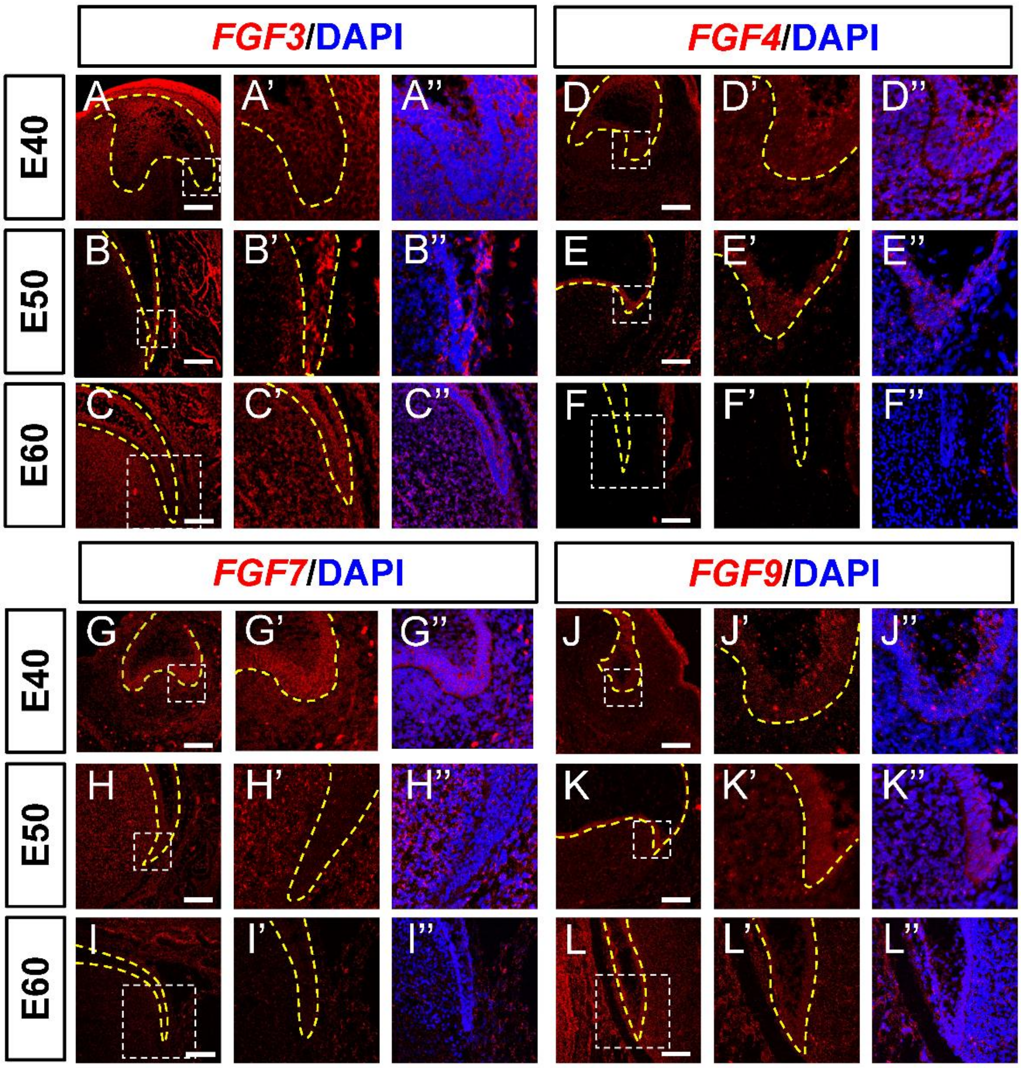

Figure S2. Dynamic expression of genes encoding FGF ligands of cervical loop during morphogenesis of DM3. (A–L) *In situ* hybridization (ISH) showing the mRNA expression of FGF ligands (red) and nuclei stained with DAPI (blue) from E40 to E60. White boxed regions in A-L are magnified in A’-L’, and DAPI stained were overlaid in A”–L”. Expression of *FGF3* (A–C) and *FGF4* (D–F) mRNA from E40 to E60; (G–I) Expression of *FGF7* mRNA from E40 to E60; (J–L) Expression of *FGF9* mRNA E40 to E60. Yellow dotted line, boundary of tooth epithelium and mesenchyme. Scale bar represents 100 μm.

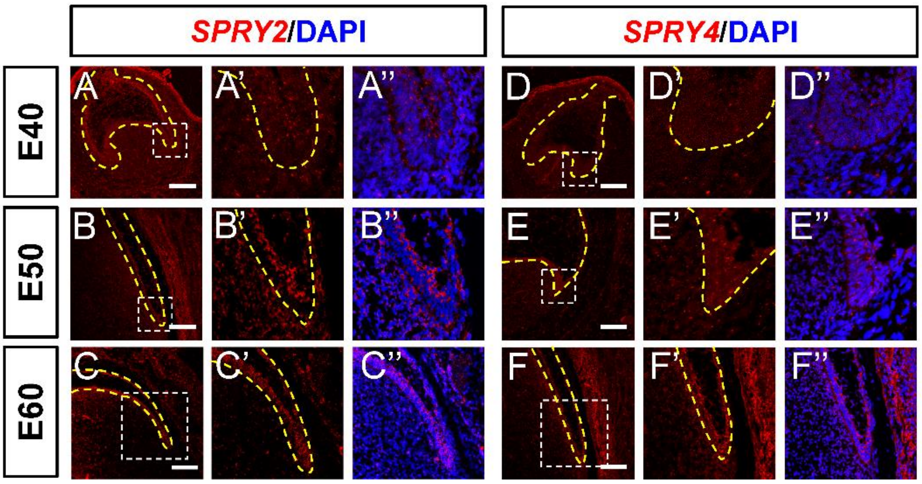

Figure S3. Dynamic expression of genes encoding FGF antagonists of cervical loop during morphogenesis of DM3.

(A–F) *In situ* hybridization (ISH) showing the mRNA expression of FGF antagonists (red) and nuclei stained with DAPI (blue) from E40 to E60. The white boxed regions in A-F are magnified in A'-F', and DAPI staining is overlaid in A''–F''. Expression of *SPRY2* (A–C) and *SPRY4* (D–F) mRNA from E40 to E60. Yellow dotted line, boundary of tooth epithelium and mesenchyme. Scale bar, 100 μm.

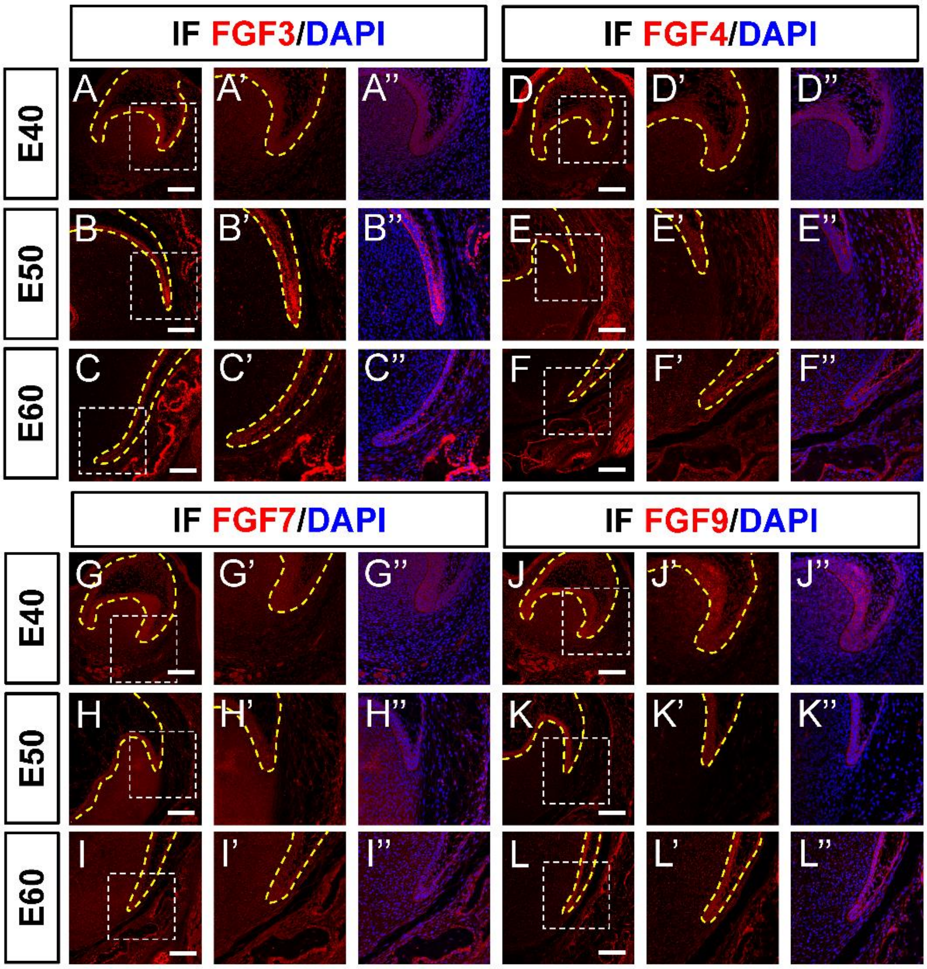

Figure S4. Protein expression of FGF ligands of cervical loop during morphogenesis of DM3.

(A–F) Immunofluorescence (IF) staining shows protein expression of FGF ligands (red) and nuclei stained with DAPI (blue) from E40 to E60. White boxed regions in A–L are magnified in A'–L', and overlaid with DAPI staining in A''–L". Expression of (A–C) FGF3, (D–F) FGF4, (G–I) FGF7 and (J–L) FGF9 from E40 to E60. Yellow dotted line, boundary of tooth epithelium and mesenchyme. Scale bar, 50 μm.

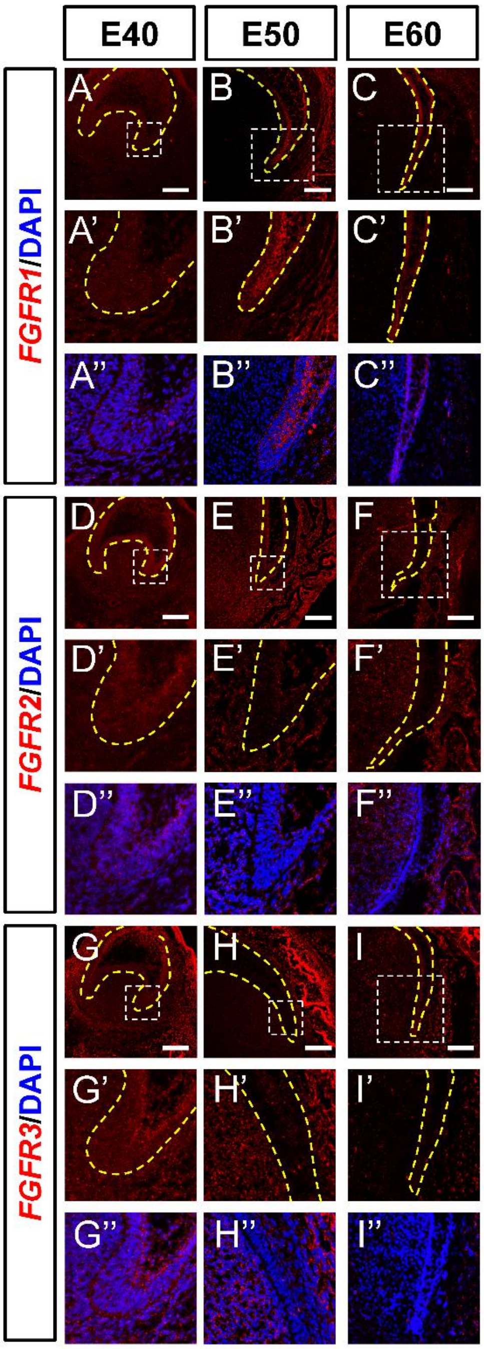

Figure S5. Dynamic expression of genes encoding FGF receptors of cervical loop during morphogenesis of DM3.

(A–I) *In situ* hybridization (ISH) shows mRNA expression of FGF receptors (red) and nuclei stained with DAPI (blue) from E40 to E60. White boxed regions in A-I are magnified in A’-I’, and DAPI staining is overlaid in A’’–I”. Expression of (A–C) *FGFR1*, (D–F) *FGFR2*, and (G–I) *FGFR3* mRNA from E40 to E60. Yellow dotted line, boundary of tooth epithelium and mesenchyme. Scale bar, 100 μm.
